# Tensins in Cancer: Integration of Their Domain Functions, Context-Dependent Regulation and Biomarker Potential

**DOI:** 10.3390/biology14081053

**Published:** 2025-08-14

**Authors:** Junyi Zheng, Hualong Zhao, Lisha Wei, Jinjun Jiang, Wenlong Xia

**Affiliations:** School of Marine and Biological Engineering, Yancheng Teachers University, Yancheng 224007, China; zhengjy@yctu.edu.cn (J.Z.); zhaohl@yctu.edu.cn (H.Z.); weils@yctu.edu.cn (L.W.); jinjunjiang2025@163.com (J.J.)

**Keywords:** tensins, protein domains, context-dependent regulation, cancer biomarkers, CTEN (TNS4)

## Abstract

Tensins (TNS1–4) are pivotal molecular scaffolds bridging the actin cytoskeleton to integrin-based adhesions, orchestrating signal transduction and governing cellular processes in cancer. Structurally, the N-terminal actin-binding domain (ABD) in TNS1–3 enables cytoskeletal regulation and interactions with regulators like the Rho GAP DLC1, while ABD-deficient TNS4 functions as a focal adhesion signal amplifier. Functionally, TNS1–3 exhibit context-dependent duality as tumor promoters or suppressors, dictated by tissue-specific microenvironments and signaling crosstalk. In contrast, TNS4 acts predominantly as an oncoprotein across carcinomas by stabilizing epidermal growth factor receptor (EGFR), driving epithelial–mesenchymal transition and invasion, and sustaining proliferation. Clinically, tensin dysregulation correlates with metastasis and poor prognosis: TNS2 serves as a diagnostic biomarker for gastrointestinal stromal tumors, aberrant TNS1/TNS3 expression predicts metastasis risk, and TNS4 is recurrently embedded in multi-gene prognostic signatures. This review synthesizes their structural basis, regulatory mechanisms, and clinical relevance, highlighting context-dependent switches and TNS4’s therapeutic potential.

## Simple Summary

Cancer metastasis drives 90% of cancer-related deaths, with aberrant cell migration playing a pivotal role. The tensin protein family (TNS1–4) governs key cellular processes in cancer progression. We integrate structural, functional, and clinical evidence regarding tensins and find that TNS1–3 play dual roles in different cancers. Conversely, TNS4 predominantly fuels cancer advancement across carcinomas. Clinically, tensins show biomarker utility: aiding diagnosis or predicting tumor aggressiveness and metastatic risk. Notably, TNS4 is incorporated into multi-gene prognostic signatures and emerges as a mechanistically rational therapeutic candidate due to its central role in oncogenic pathways. Deeper understanding of tensin biology may enable improved diagnostic/prognostic strategies and pave the way for targeted therapies.

## 1. Introduction

Cancer is the second leading cause of mortality worldwide [1]. Its complex nature is encapsulated by the six established hallmarks, which include sustaining proliferative signaling, evading growth suppressors, resisting cell death, enabling replicative immortality, inducing angiogenesis, and activating invasion and metastasis [2]. Among these, metastasis stands out as the predominant cause of cancer lethality, accounting for approximately 90% of cancer-related deaths [3]. A key driver of metastasis is aberrant cell migration. While cell migration is a vital characteristic essential for physiological processes like embryonic development, immune responses, and wound healing [4,5,6], it becomes a detrimental hallmark when co-opted by cancer cells to facilitate tumor dissemination [2]. Metastatic cells typically exhibit significantly enhanced migration rates compared to their counterparts in primary tumors [7], underscoring the critical need to understand the mechanisms governing cancer cell motility.

Cell migration fundamentally relies on dynamic interactions between cells and the extracellular matrix (ECM). These interactions are primarily mediated by specialized adhesion complexes, which are intricate molecular assemblies composed of integrins, talin, kindlin, tensin, and numerous other proteins. Tensin was first identified in 1991 as an actin-binding protein featuring an Src homology 2 (SH2) domain and tyrosine phosphorylation sites [8], and it emerged as a potential linker between signal transduction pathways and the cytoskeleton. Mammals express four distinct tensin paralogs, namely tensin 1 (TNS1), tensin 2 (TNS2/TENC1/C1-TEN), tensin 3 (TNS3), and tensin 4 (TNS4/CTEN) [9,10,11].

Given their critical localization within adhesion complexes and role in modulating cell adhesion and migration, tensins are pivotal factors in cancer progression. This review synthesizes our current understanding of the four human tensins, covering their molecular structure, cellular functions, involvement in key signaling pathways, context-dependent roles in tumorigenesis and cancer metastasis, and clinical significance.

## 2. Structural Domains of Tensins and Their Functional Implications

**Figure 1:**
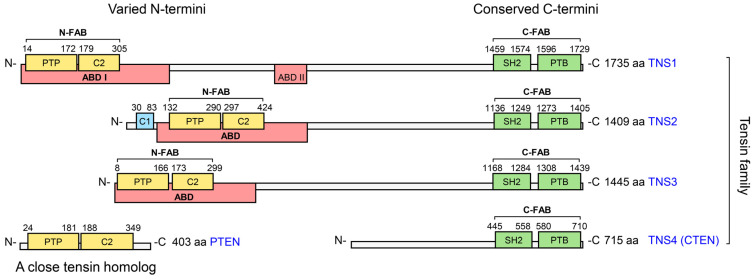
Domain structures of human TNS1–4 and a close tensin homolog PTEN.

The four human tensins exhibit high sequence similarity and share several conserved domains (Figure 1). All four tensins harbor conserved C-terminal Src homology 2 (SH2), and phosphotyrosine-binding (PTB) domains, which are tandemly arranged to their C termini [12]. TNS1, TNS2, and TNS3 also possess the N-terminal protein tyrosine phosphatase (PTP) and protein kinase C conserved region 2 (C2) domains, which are lost in TNS4 [12]. Uniquely, TNS2 features a protein kinase C conserved region 1 (C1) domain localized to the N-terminus, which is not shared by other tensins [13]. Functionally, all four tensins localize to focal adhesions through focal adhesion binding (FAB) domains, while TNS1–3 also bind actin via actin-binding domains (ABDs), which reside N-terminally and are again lost in TNS4. These domains confer dual functionalities: bridging the cytoskeleton to the ECM and facilitating signal transduction. Notably, the absence of the N-terminal ABD and PTP-C2 in TNS4 highlights a major evolutionary divergence within the family, potentially linked to its distinct roles in cancer progression.

### 2.1. ABD

TNS1–3 possess conserved N-terminal ABDs responsible for actin binding, which is absent in TNS4 [11]. Initial studies using baculoviral-expressed recombinant chicken TNS1 identified its actin filament binding ability and defined two distinct ABDs [14]. ABD-I, located near the N-terminus, binds to the side of actin filaments, while ABD-II, situated in the middle of the protein, interacts with the barbed end of actin filaments and modulates actin polymerization rates [14]. The ABDs of human tensins are assigned based on high sequence similarities, and notably, only the N-terminal ABD-I is conserved in TNS2 and TNS3. Beyond actin binding, the ABDs of TNS1 and TNS3 also interact with the Rho GTPase-activating protein (GAP) Deleted in Liver Cancer 1 (DLC1), regulating Ras homolog family member A (RhoA) activity [15,16]. Additionally, the TNS3 ABD interacts with Dock5, a guanine nucleotide exchange factor (GEF) for Rac GTPase, influencing Rac activity in osteoclasts [17]. The loss of ABD in TNS4 represents a key evolutionary adaptation. Unlike TNS1/3, which anchor actin to mechanically stabilize cells, TNS4’s ABD-free structure may favor rapid signal transduction, a trait potentially advantageous for cancer cell migration.

### 2.2. N-Terminal FAB

Tensins localize to and interact with focal adhesions through FAB domains. TNS1–3 contain both N-terminal and C-terminal FAB domains, while TNS4 possesses only the C-terminal FAB domain. The N-terminal FAB domains of TNS1–3 reside within their ABDs and include conserved PTP and C2 domains. TNS2 features a unique protein C1 domain localized to the N-terminal, just next to its PTP domain, a characteristic not shared by other tensins, though its function remains unclear [13]. The exclusive presence of a C1 domain in TNS2 suggests a late evolutionary acquisition.

#### 2.2.1. PTP Domain

The PTP domains of TNS1–3 belong to the cysteine-based PTP family, identified by a conserved CxxxxxR motif. However, critical cysteine residues are absent within the PTP motif of TNS1, indicating that it is catalytically inactive [18]. Conversely, the TNS2 and TNS3 PTP domains retain the essential cysteine residue. Importantly, TNS2 exhibits demonstrable PTP activity, capable of dephosphorylating the pTyr-612 residue of insulin receptor substrate-1 (IRS-1). This dephosphorylation reduces IRS-1 stability and subsequently inhibits activation of the protein kinase B (Akt) and AMP-activated protein kinase (AMPK) pathways [19,20]. But the specific substrate for TNS3 PTP has not yet been identified. The retention of catalytic activity in TNS2, while it is inactive in TNS1 and uncharacterized for TNS3, implies evolutionary pressure for phosphatase-dependent signaling.

#### 2.2.2. C2 Domain

The TNS1 C2 domain encompasses a 299KVXF302 motif that facilitates its interaction with serine/threonine protein phosphatase 1α (PP1α) and recruits PP1α to focal adhesions [21,22]. Furthermore, this domain mediates TNS1’s interaction with the sterile alpha motif (SAM) of DLC1 and modulates Rho GTPase activity within cells [15]. Similarly, the TNS3 C2 domain directly interacts with the DLC1 SAM domain and promotes DLC1 activation. Activated DLC1 subsequently inactivates RhoA and reduces cell migration [16,23].

### 2.3. C-Terminal FAB

All four human tensins are associated with focal adhesions via their C-terminal FAB domains, characterized by tandemly arranged SH2 and PTB domains. As both domains are critical for signal transducing, the conserved C-terminal SH2-PTB tandem represents an evolutionarily stable signaling scaffold [24,25,26,27].

#### 2.3.1. SH2 Domain

The SH2 domain plays crucial roles in tensin-mediated signal transduction by binding phosphotyrosine (pTyr) and lipids [24]. Specifically, tensin SH2 domains recruit pTyr-containing proteins, including epidermal growth factor receptor (EGFR), hepatocyte growth factor receptor (MET), Axl, Src, focal adhesion kinase (FAK), and p130Cas (BCAR1). These interactions trigger signaling cascades mediated by protein tyrosine kinases [8,10,28,29]. Notably, unlike canonical SH2 domains, which exclusively bind pTyr sites, the SH2 domains of tensins bind DLC1 independently of tyrosine phosphorylation [30,31,32,33].

#### 2.3.2. PTB Domain

The PTB domains of tensins directly bind the cytoplasmic tails of integrin subunits β1, β3, β5, and β7 through the NPXY motifs [25,26,27], thereby linking actin filaments to focal adhesion sites. Importantly, the PTB domains of TNS1 and TNS2 also bind DLC1 [30,34,35]. The absence of PTB-DLC1 binding in TNS3/4 reveals a subfamily-specific co-option, potentially fine-tuning Rho GTPase signaling in tissue contexts prone to malignancy.

### 2.4. Structural and Functional Divergence

Phylogenetic analyses indicate that TNS4 represents a late evolutionary branch within the family, supported by its exclusive presence in mammals and high sequence conservation across species [36]. This late emergence coincides with significant structural divergence: TNS4 lacks the conserved N-terminal region (including ABD, PTP, and C2 domains) present in TNS1–3. Notably, PTEN, a tensin homology, contains domains structurally similar to the PTP and C2 domains of TNS1–3 but lacks other tensin-specific regions (Figure 1) [37]. This observation has led to the hypothesis that PTEN and TNS4 may have originated from the splitting of a common ancestral gene [38]. Similarly, the unique presence of the C1 domain in TNS2 likely reflects a lineage-specific evolutionary acquisition.

These structural differences underpin functional diversification among tensins. The C-terminal SH2-PTB tandem is evolutionarily conserved across all four family members, serving as a stable scaffold for integrin binding, focal adhesion localization, and interactions with key signaling partners such as EGFR, MET, Axl, Src, FAK, and p130Cas [2,3,4,5,6,7,8,9,10,11,12,13,14,15,16,17,18,19,20,21,22,23,24,25,26,27]. In contrast, the N-terminal ABDs exclusive to TNS1–3 enable actin cytoskeleton linkage and mechanotransduction. The absence of N-terminal domains in TNS4 likely underlies its functional divergence toward amplified signal transduction at focal adhesions, a mechanism frequently dysregulated in cancer, which thereby drives disease progression.

## 3. Regulatory Roles of Tensins in Cellular and Physiological Processes

Building on their domain-specific functionalities, tensins critically regulate fundamental cellular processes through focal adhesion dynamics. These include cell adhesion, migration/invasion, proliferation, and mechanotransduction—processes whose dysregulation directly contributes to tumorigenesis and metastatic progression.

### 3.1. Cell Dhesion

Cell–cell and cell–ECM interactions fundamentally depend on adhesion, which is crucial for regulating cell migration, proliferation, and mechanotransduction. Cell–matrix adhesions are primarily mediated by integrin-based structures, including focal adhesions, hemidesmosome, and podosomes, existing in different cell types [39]. As pivotal adhesion mediators, vertebrate integrins comprise 18 α and 8 β subunits, forming 24 distinct heterodimers [40]. These integrins, together with adaptor proteins, assemble into adhesion complexes linking cells to the ECM [41]. Fibroblasts adhering to the ECM initially form nascent adhesions that progress into dot-like focal complexes, later mature into larger focal adhesions, and ultimately elongate into fibrillar adhesions [42].

Tensins localize to adhesion complexes but exhibit distinct distributions: TNS2 predominantly to focal adhesions, TNS3 primarily to fibrillar adhesions, and TNS1 at both sites [12]. Specifically, TNS1 and TNS3 play critical roles in the maturation of fibrillar adhesions, which are essential for matrix remodeling [43]. Supporting this role, silencing TNS1 or TNS3 in AMPK α1/α2-deficient fibroblasts reduced fibrillar adhesion formation and fibronectin fibrillogenesis [43]. Conversely, TNS2 knockdown significantly diminished the capacity of human foreskin fibroblasts to contract 3D collagen gels without affecting fibronectin fibrillogenesis, indicating that TNS2 specifically functions in collagen remodeling rather than general adhesion complex formation [44]. TNS4, which lacks the N-terminal ABD, interacts with integrin β1 and predominantly localizes to focal adhesions [26]. In MCF10A cells, TNS4 displaced TNS3 from the integrin β1 cytoplasmic tail, inducing actin fiber disassembly and impaired cell adhesion [26]. However, in RWPE-1 nonmalignant prostatic epithelial cells, ΔNp63α (the predominant p63 isoform) enhanced adhesion by promoting TNS4 expression [45], indicating a context-dependent effect of TNS4 on adhesion.

### 3.2. Cell Migration and Invasion

Tensins exhibit diverse and context-dependent effects on cell migration and invasion, with TNS1 predominantly demonstrating pro-migratory functions. For example, GFP-TNS1 overexpression enhanced the migration of the human embryonic kidney (HEK) cell HEK293 [9]. Endothelial cells derived from TNS1-knockout mice or subjected to TNS1 siRNA silencing exhibited reduced migration [15]. Mechanistically, TNS1 facilitates RhoA activity by interacting with DLC1 via its C2, SH2, and PTB domains [15] and enhances cell migration by linking inwardly translocating actin cytoskeletons to phosphorylated Tyr-p130Cas at focal adhesions [46]. Furthermore, TNS1 drives the growth and metastasis of non-small-cell lung cancer (NSCLC) cells via the Akt/mTOR/RhoA pathway [47]. Conversely, TNS1 can suppress migration/invasion: miR-548j enhanced invasion and metastasis of MCF7 breast cancer cells by targeting TNS1 and activating Cdc42 [48]. MicroRNA-mediated downregulation of TNS1 is associated with increased migration, invasion, and metastasis in colon adenocarcinoma, bladder cancer, and lung cancer [49,50,51], supporting its potential tumor-suppressive role in specific contexts.

TNS3 also acts primarily as a migration/invasion inhibitor. For instance, epidermal growth factor (EGF) treatment downregulated TNS3 in MCF10A cells, enhancing migration [26]. Consistently, stable TNS3 expression in HEK293 cells suppressed migration and matrix invasion, while TNS3 knockdown increased migration in human cancer cells [52]. In glioblastoma, Musashi-1 (MSI1), an RNA-binding protein, inhibited TNS3 translation by binding its mRNA 3’UTR, promoting migration [53]. Additionally, elevated TNS3 mRNA levels correlated with decreased migration and invasion in malignant thyroid cell lines [54,55], TNS3 promoter hypermethylation was linked to renal cell carcinoma (RCC) metastasis [56], and mixed-lineage leukemia 3 (MLL3)-activated TNS3 suppressed U2OS cell migration [57]. In contrast, SUV420H2 downregulated TNS3 and suppressed invasiveness in MDA-MB-231 cells [58]. Furthermore, silencing of TNS3 in human tonsil-derived mesenchymal stem cells significantly reduced cell migration [59], indicating a potential context-dependent pro-migratory function for TNS3.

Evidence for TNS2 remains limited and context-dependent. Ectopic TNS2 expression inhibited HEK293 cell migration [60], whereas TNS2 knockdown impaired integrin internalization and decreased the invasiveness of Rab25-transfected A2780 ovarian cancer cells [61]. These observations suggest that TNS2 has both inhibitory and promotive effects.

In stark contrast, TNS4 consistently promotes migration and invasion across cancers. For instance, TNS4 facilitates migration, invasion, and epithelial–mesenchymal transition (EMT) in colorectal cancer cells, potentially through upregulation of integrin-linked kinase (ILK), FAK, and Src [62,63,64,65,66]. In lung adenocarcinoma cells, TNS4 induces TGF-β1 expression to promote EMT [67]. In breast cancer, it is upregulated by EGF, displacing TNS3 from actin to promote migration [16,26]. Furthermore, TNS4 promotes migration in liver, pancreas, and skin cancer cells [63,68,69], collectively underscoring its oncogenic role in enhancing cell motility.

### 3.3. Cell Proliferation

Tensins critically regulate the proliferation of both normal and cancerous cells, with distinct effects: TNS1, TNS3, and TNS4 generally act as positive regulators, while TNS2 functions as a negative regulator.

Supporting TNS1’s positive regulatory role, endothelia cells from TNS1-knockout mice or mice with TNS1 siRNA silencing exhibited decreased proliferation [15]. Furthermore, TNS1 transfection into NSCLC cells elevated Akt/mTOR and RhoA activity, correlating with enhanced proliferation [47]. TNS1 knockdown also diminished proliferation in SW620 colon cancer cells [70] and suppressed the PI3K/Akt/mTOR pathway, decreasing proliferation in U937 and HL60 acute myeloid leukemia cells [71].

In line with TNS1, TNS3 knockdown using siRNA in tonsil-derived mesenchymal stem cells upregulated cyclin-dependent kinase (CDK) inhibitors p16 and p21, reducing proliferation [72]. Moreover, silencing TNS3 with the histone deacetylase inhibitor LMK-235 inhibited esophageal squamous cell carcinoma (ESCC) cell proliferation in vitro and in vivo [73].

TNS4 also positively regulates proliferation. It interacts with phosphorylated MET via its SH2 domain, enhancing survival and proliferation by stabilizing MET [29]. Additionally, epidermal growth factor (EGF)-induced extracellular regulated protein kinase 1/2 (ERK1/2) upregulates TNS4, promoting proliferation and migration in hepatocellular carcinoma [68]. Consistent with this, TNS4 silencing increased the accumulation of CDK inhibitors p21 and p27, reducing proliferation in RWPE-1 prostate epithelial cells [74]. Furthermore, elevated TNS4 levels were detected in lung adenocarcinoma (LUAD) tissues, while in vitro TNS4 knockdown resulted in a decrease in both cell proliferation and migration [75].

In contrast, TNS2 inhibits proliferation. Its overexpression in HEK293 cells reduced proliferation and survival [60], while silencing TNS2 upregulated Akt, mitogen-activated protein kinase kinase (MAPKK, also termed as Mek), and insulin receptor substrate 1 (IRS1) activities, promoting proliferation in HeLa and A549 cells [13]. These findings indicate that TNS2 negatively regulates cell proliferation, potentially by suppressing the IRS-1, Akt, and MEK-ERK signaling pathways.

### 3.4. Mechanotransduction

Tensins act as mechanosensors by localizing to adhesion complexes and mediating cellular responses to mechanical forces, with TNS1 being the most comprehensively characterized. In fibroblasts, AMPK knockout upregulated TNS1, enabling its binding to β1-integrins to facilitate fibrillar adhesion formation and integrin-dependent processes [43]. Conversely, TNS1 silencing significantly reduced the fibrillar adhesion length on stiffness-gradient gels [76]. In cancer-associated fibroblasts (CAFs), increased substrate rigidity enhanced the interaction between Src-phosphorylated TNS1 and Hic-5, further promoting fibrillar adhesion formation [77]. These findings collectively highlight TNS1’s critical role in stiffness-induced adhesion maturation. Additionally, TNS1 facilitates migration by coupling inwardly translocating actin to phospho-Tyr-p130Cas at focal adhesions [46].

TNS3, similarly to TNS1, was upregulated following AMPK knockout, and its silencing disrupted integrin binding and activation, hindering fibrillar adhesion formation [43]. In contrast, TNS4 lacks the ABD and does not directly engage in actin filament dynamics. Nevertheless, it rapidly accumulates along tension-bearing keratin fibers during epithelial cell stretching [78], suggesting a role in keratin-based mechanotransduction.

## 4. Regulatory Roles of Tensins in Signaling Pathway Crosstalk

Beyond mediating discrete cellular processes, tensins serve as pivotal signaling scaffolds that orchestrate intricate crosstalk between key oncogenic pathways. Through their multi-domain architecture, tensins integrate mechanical cues from focal adhesions with biochemical signals from receptors (e.g., EGFR, integrins), thereby modulating pathway activities whose dysregulation underpins cancer hallmarks.

### 4.1. EGFR Signaling Pathways

The binding of EGF to EGFR, a receptor tyrosine kinase, initiates signaling pathways regulating tissue development and homeostasis. This signaling can also be activated by alternative ligands, resulting in receptor dimerization, autophosphorylation, and activation of downstream molecules mediating proliferation, migration, and differentiation [79]. Notably, EGFR upregulation in cancer frequently leads to hyperactivation of pro-oncogenic pathways like RAS-RAF-MEK-ERK MAPK and AKT-PI3K-mTOR [80].

**Figure 2:**
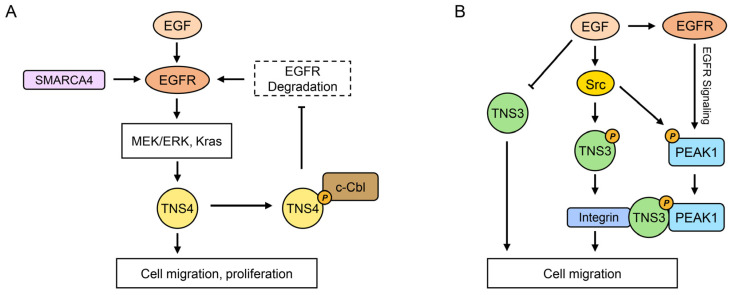
Crosstalk of EGFR signaling pathways with TNS3 and TNS4. (**A**) TNS4 functions as both a downstream effector and upstream regulator of EGFR; (**B**) TNS3 indirectly engages in EGFR signaling through PEAK1. The “*p*” refers to phosphorylation of proteins.

Among the tensin family, TNS4 exhibits the most extensively documented involvement in EGFR signaling, functioning both as a downstream effector and an upstream regulator (Figure 2). In MCF10A breast epithelial cells, EGF treatment upregulates TNS4 mRNA while downregulating TNS3 mRNA [26]. Although the mechanism for TNS3 downregulation remains unclear, TNS4 induction is mediated by EGFR-activated Erk, evidenced by its complete inhibition by the Mek inhibitor U0126 [26]. This regulatory mechanism is corroborated in hepatocellular carcinoma (HCC) cells, where EGF-induced ERK1/2 activity drives TNS4 upregulation [68]. Furthermore, in colorectal cancer cells, the EGFR/Kras pathway specifically upregulates TNS4, though without affecting TNS3 [81,82], and in HeLa cells, the EGFR-activated RAF/MEK/ERK pathway facilitates p300 binding to the TNS4 promoter, enhancing its expression [83]. These findings collectively suggest that EGF-induced hyperactivation of EGFR/ERK signaling significantly contributes to the frequent overexpression of TNS4 across cancers. Additionally, SMARCA4 recruited by PRMT1-mediated H4R3me2a enhances EGFR signaling and TNS4 expression in colorectal cancer, with elevated PRMT1 or SMARCA4 levels positively correlating with increased EGFR and TNS4 expression and decreased overall survival [84]. Moreover, a mutant SMARCA4R enhances the SWI/SNF complex’s ATPase activity, facilitating chromatin remodeling, which reinforces EGFR and TNS4 transcriptional expression, promoting proliferation in colorectal cancer cells and patient-derived tumor organoids [85]. Collectively, these studies establish TNS4 as a key downstream effector in EGFR signaling pathway.

Beyond its role as an effector, TNS4 reciprocally regulates EGFR stability. EGFR signaling is tightly controlled by ligand binding, which activates the receptor but also triggers suppression mechanisms, including ubiquitin-dependent degradation primarily mediated by the E3 ubiquitin ligase c-Cbl [86]. c-Cbl is associated with tyrosine-phosphorylated EGFR, promoting its ubiquitination and subsequent internalization and lysosomal degradation [86,87]. Disruption of this negative feedback mechanism may contribute to tumorigenesis. Notably, TNS4 attenuates ligand-induced EGFR degradation [88]. Mechanistic studies on HEK293T cells and cancer cell lines (SW480 colon, A549 lung, 5637 bladder) show that the TNS4 SH2 domain interacts with EGFR-phosphorylated c-Cbl, sequestering it away from activated EGFR. This interaction reduces EGFR degradation, resulting in sustained EGFR pathway activation, which is implicated in tumor progression [88]. Further supporting this upstream regulatory role, miR-1224-5p inhibits TNS4 expression in ESCC cells, consequently reducing EGFR protein levels without affecting mRNA [89]. Importantly, silencing TNS4 mitigated the effects of miR-1224-5p loss on EGFR upregulation and signaling activation, as well as inhibiting in vitro proliferation, colony formation, migration, invasion, and in vivo tumor growth [89]. These findings highlight TNS4 as an upstream regulator of EGFR signaling. Conversely, in prostate cancer and related cell lines, TNS4 is downregulated [11] and exhibits a context-specific function by downregulating EGFR expression [90], indicating a different role in this context.

The associations of other tensins with EGFR signaling are less prominent compared to TNS4. The literature on TNS3 and EGFR is significantly less extensive, and mammalian TNS1 and TNS2 show no reported associations. Notably, TNS3 is phosphorylated upon EGF induction [10]. Although Src, rather than EGFR, primarily phosphorylates TNS3 in response to EGF, EGF facilitates the TNS3–EGFR interaction [10]; however, its functional significance remains undetermined. Additionally, in mouse epithelial GE11 cells, TNS3 connects to PEAK1 and integrins through its SH2 domain binding to PEAK1 [91]. Given that PEAK1 serves as a scaffolding protein involved in later EGFR-mediated signaling responses [92], this suggests that TNS3 may indirectly engage in EGFR signaling through PEAK1 (Figure 2). Therefore, while TNS4 plays a central and bidirectional role, other tensins show limited or indirect connections, collectively functioning as both regulators and effectors facilitating crosstalk between EGFR and other pathways.

### 4.2. Rho GTPase Signaling Pathways

Tensins regulate cell proliferation, adhesion, and migration through mechanisms critically involving small GTPases from the Rho family, which are pivotal modulators of actin dynamics, with RhoA, Rac1, and Cdc42 being the most extensively studied members [93]. These GTPases switch between active GTP-bound and inactive GDP-bound states, regulated by GEFs, which promote activation by facilitating GTP binding [94], and GAPs, which promote inactivation by enhancing GTP hydrolysis [95]. Complex interactions among Rho GTPases, GEFs, and GAPs orchestrate cellular processes like adhesion and migration, often characterized by reciprocal regulation. For example, Rac1 activation inhibits RhoA during the initial adhesion stages, whereas later RhoA activation reduces Rac1 activity [96].

**Figure 3:**
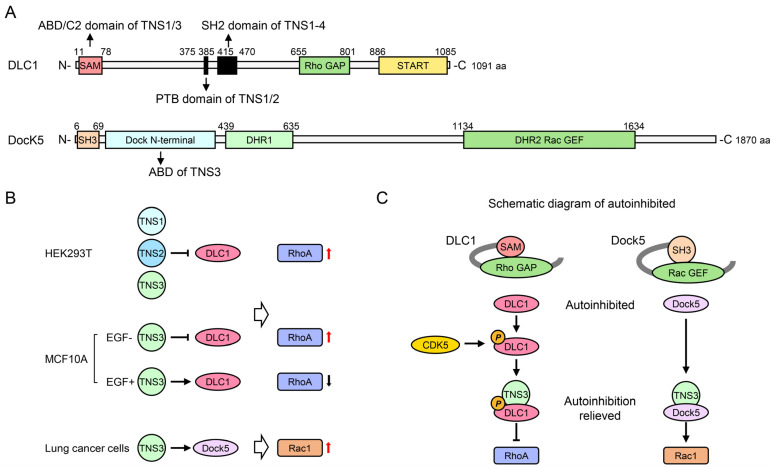
Roles of TNS1–3 in Rho GTPase signaling pathways. (**A**) Sehematic of domains of DLC1 and Dock5, black arrows indicate binding partners and binding sites; (**B**) cell context-dependent regulation of DLC1 and Dock5 by TNS1–3, “EGF-/EGF+” refer to without/with EGF induction, “⬆/⬇” refer to up/down-regulation of protein activitiies respictively; (**C**) TNS3 relieves autoinhibition of DLC1 (inhibiting RhoA) and Dock5 (activating Rac1). The “*p*” refers to phosphorylation of proteins.

DLC1, a Rho GAP frequently lost in cancers, serves as a central link between tensins and Rho signaling. Its re-expression inhibits cancer cell proliferation [97]. DLC1 reduces RhoA activity by promoting GTP hydrolysis, thereby regulating adhesion, morphology, and migration via actin dynamics and focal adhesion turnover [98]. Crucially, all four tensins bind DLC1 via their SH2 domains independently of DLC1 tyrosine phosphorylation (Figure 3) [30,31,32,33]; however, they utilize distinct secondary binding sites. TNS4 binds solely via its SH2 domain, while TNS2 possesses an additional binding site within its PTB domain [34,35]. For TNS3, the secondary site resides in its N-terminal C2 domain rather than the PTB domain [16,23]. In the case of TNS1, the C2, SH2, and PTB domains are essential for its interaction with DLC1 [15,30].

The functional consequences of these interactions also vary significantly. An investigation into HEK293T cells, which lack endogenous TNS1 and DLC1, showed that TNS1 overexpression alone did not influence RhoA activity; however, co-expression with DLC1 and TNS1 counteracted DLC1’s effect on RhoA activity [15], indicating that TNS1 modulates RhoA activity in a DLC1-dependent manner. Similar assays for TNS2, TNS3, and TNS4 demonstrated that only TNS2 or TNS3 expression increased RhoA activity in the presence of DLC1, whereas TNS4 did not [15]. This implies that the N-terminal region absent in TNS4 is necessary to inhibit DLC1’s Rho GAP activity. Consequently, the differential regulation of DLC1 by tensins translates into diverse effects on cell adhesion and migration.

TNS1 primarily promotes RhoA signaling and exhibits oncogenic potential in this context (Figure 3). Silencing TNS1 suppressed RhoA activity in human umbilical vascular endothelial cells, an effect that is reversible by subsequent DLC1 knockdown, correlating with reduced proliferation and migration [15]. Furthermore, a mutation disrupting the TNS1 SH2–DLC1 interaction decreased invasion in MDA-MB-231 cells [22]. Notably, a mutation in the TNS1 C2 domain, which weakens its interaction with PP1α, also impaired DLC1 interaction but unexpectedly promoted invasion [22], suggesting that PP1α binding is essential for TNS1’s ability to inhibit invasion.

TNS2 also enhances RhoA activity (Figure 3). TNS2 is enriched in dynamic focal adhesions at the leading edge of human foreskin fibroblasts (HFFs); its knockdown diminished their capacity to contract three-dimensional collagen gels and reduced Rho activity [44]. Significantly, DLC1 knockdown restored the contraction ability of TNS2-deficient fibroblasts [44], highlighting a mechanism where TNS2 regulates Rho activity and collagen contraction by modulating the DLC1 Rho GAP function.

TNS4 interacts with DLC1 without altering its intrinsic GAP activity. Nevertheless, the TNS4–DLC1 interaction is crucial for DLC1’s tumor-suppressive activity by enabling its localization to focal adhesions [33].

TNS3 exhibits context-dependent effects on RhoA (Figure 3). In DLC1-expressing cells, like HFFs, H157/H1703 NSCLC cells, and HEK293T cells expressing exogenous DLC1, functional studies suggest that TNS3 acts as an inhibitor of DLC1, thereby enhancing RhoA activity, similarly to TNS1 and TNS2 [15,44]. Contrarily, in MCF10A cells, TNS3 was found to inhibit RhoA activity [16]. EGF treatment downregulated TNS3 while upregulating TNS4, which is associated with increased active RhoA levels; furthermore, silencing TNS3 significantly activated RhoA and promoted cell migration [16]. This apparent contradiction is resolved by considering DLC1 autoinhibition mechanisms. DLC1’s SAM domain serves as an autoinhibitory switch; its deletion significantly enhances DLC1-mediated RhoA inactivation [99]. Specifically, in MCF10A cells, TNS3 activates DLC1’s Rho GAP function by disrupting the autoinhibitory interaction between the SAM and Rho GAP domains [16]. Therefore, the cellular context, particularly the mechanisms governing DLC1 autoinhibition, dictates whether TNS3 enhances or suppresses RhoA activity.

DLC1 autoinhibition involves multiple mechanisms. One involves the serine/threonine kinase CDK5, which phosphorylates four serine residues in the DLC1 region N-terminal to the Rho-GAP domain. In its unphosphorylated state, this N-terminal region interacts with the Rho-GAP domain, maintaining DLC1 inactivity [100]. Conversely, CDK5 phosphorylation enables this region to bind the C-terminal region of TNS3, thereby enhancing DLC1 Rho-GAP activity [100]. Importantly, the basal activity of CDK5 has been demonstrated to be very low in MCF10A cells [100], suggesting minimal CDK5-mediated decrease in autoinhibition. Furthermore, SAM domain-mediated autoinhibition appears to be linked to EGF signaling; without EGF induction, silencing TNS3 in MCF10A cells reduced Rho GTP levels [100], consistent with TNS3’s RhoA-enhancing role observed in other DLC1-positive cells.

Beyond RhoA, TNS3 also regulates Rac1 activity (Figure 3). In MCF10A cells, EGF stimulation triggers threonine phosphorylation of TNS3 and PTEN, which induces partner swapping between TNS3-DLC1 and PTEN-PI3K complexes, forming new TNS3-PI3K and PTEN-DLC1 assemblies [101]. The TNS3-PI3K complex then translocate to the cell’s leading edge to activate Rac1, while PTEN-DLC1 moves posteriorly to locally activate RhoA [101]. Additionally, TNS3 activates Rac1 through its interaction with the Rac1 GEF Dock5 in osteoclasts [17]. Co-expression of TNS3 and Dock5 in HEK293T cells significantly enhanced Rac activation by Dock5, whereas TNS3 expression alone had no effect on Rac activity [17]. Mechanistically, Dock5 is autoinhibited by its N-terminal SH3 domain, while the binding of TNS3 ABD to Dock5’s N-terminal region lessens this autoinhibition [17]. Collectively, these findings illustrate how tensins orchestrate cell adhesion and migration by precisely fine-tuning Rho GTPase signaling pathways through direct interactions and modulation of key regulators like DLC1 and Dock5.

### 4.3. PI3K/Akt/mTOR Signaling Pathway

The phosphatidylinositol 3-kinase (PI3K), protein kinase B (Akt), and mechanistic target of rapamycin (mTOR) constitute a complex and critical signaling axis governing proliferation, survival, metabolism, motility, and stress responses [102,103]. Upon signal induction, activated receptor tyrosine kinases (RTKs) recruit PI3K to the plasma membrane to convert PIP2 into PIP3. This lipid second messenger activates Akt via coordinated phosphorylation by PDK1 and mTORC2 [102]. Activated Akt then phosphorylates TSC2, preventing its suppression of mTORC1. Subsequently, mTORC1 activation triggers a negative feedback loop mediated by S6K that downregulates Akt activity [102]. Additionally, mTORC1 is regulated independently through the RTK-initiated RAS/RAF/MEK/ERK cascade [104].

**Figure 4:**
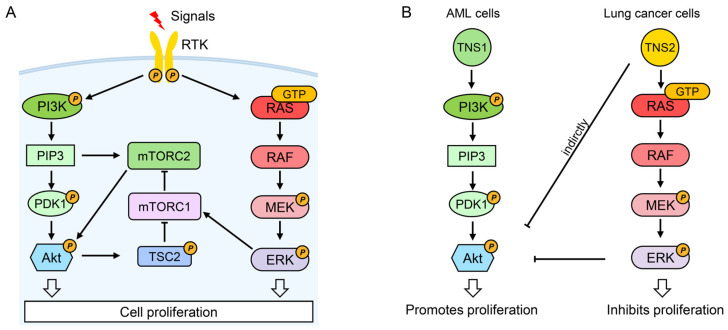
Roles of TNS1 and TNS2 in PI3K/Akt/mTOR signaling pathway. (**A**) Schematic of crosstalk between PI3K/Akt/mTOR and RAS/RAF/MEK/ERK signaling pathways; (**B**) differential regulation of PI3K/Akt/mTOR signaling by TNS1 and TNS2. The “*p*” refers to phosphorylation of proteins.

Hyperactivation of this pathway is frequent in cancer, prompting significant therapeutic interest. Again, tensin family members exert opposing regulatory effects on this pathway. TNS1 promotes PI3K/Akt/mTOR signaling (Figure 4). In acute myeloid leukemia (AML), prazosin inhibited viability and induced cell cycle arrest in U937 and HL60 cell lines, reducing phosphorylated Akt (p-Akt) and mTOR (p-mTOR) [71]. Significantly, prazosin downregulated TNS1 expression, and silencing TNS1 using siRNAs similarly inhibited p-Akt and p-mTOR in these cells [71], underscoring TNS1’s role in promoting pathway activity and proliferation. Consistent with this, TNS1 overexpression in A549 NSCLC cells elevated p-Akt and p-mTOR levels and enhanced proliferation, while an Akt inhibitor reduced pathway activation and inhibited TNS1-induced cell growth [47].

Conversely, TNS2 inhibits PI3K/Akt/mTOR signaling (Figure 4). Silencing TNS2 upregulated Akt, Mek, and IRS1 activities, promoting proliferation and colony formation in HeLa and A549 cells [13]. Specifically, it increased phosphorylation levels of IRS1, Akt, Mek, Erk, and IRS1 protein levels, and application of a PI3K inhibitor suppressed the TNS2 knockdown-induced phosphorylation and protein levels of IRS1 in A549 cells [13], suggesting that TNS2 inhibits cancer cell proliferation by downregulating the PI3K/Akt/mTOR pathway, possibly through activating the RAS/RAF/MEK/ERK cascade.

Clinical observations also align with these functional roles. TNS1 expression is significantly elevated in NSCLC tissues and correlates with poor prognosis [47], whereas TNS2 mRNA is significantly downregulated in head and neck, esophageal, breast, lung, liver, and colorectal cancers [13]. These findings imply that dysregulation of tensins contributes to PI3K/Akt/mTOR pathway hyperactivation in cancer.

### 4.4. Wnt and YAP Signaling Pathways

The Wnt signaling pathway represents an intercellular signaling cascade initiated by secreted, lipid-modified Wnt family ligands. Crucially, this pathway regulates cell development, proliferation, and migration and is strongly implicated in cancer. Its fundamental mechanism involves Wnt ligand binding to specific receptors, like Frizzled (FZD) and LRP5/6, on target cells, thereby triggering intracellular signal transduction. Wnt signaling is categorized into the canonical (β-catenin-dependent) and the non-canonical (β-catenin-independent) branches [105]. The canonical pathway primarily regulates cell proliferation, while the non-canonical branch encompasses the Wnt/planar cell polarity (PCP) and Wnt/calcium (Wnt/Ca^2+^) pathways, which can antagonize canonical signaling under specific circumstances [105].

**Figure 5:**
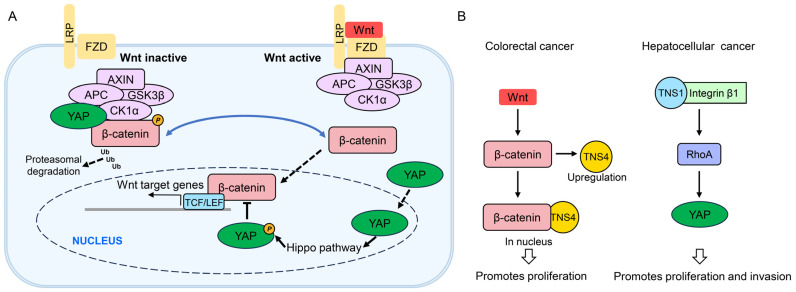
Roles of TNS1 and TNS4 in Wnt and YAP signaling pathways. (**A**) Schematic of crosstalk between Wnt and YAP signaling pathways, *“p”* refers to phosphorylation of β-catenin, the blue arrow indicates the switch between phosphorylated and unphosphorylated state, black dashed arrows indicate the translocation of proteins; (**B**) regulation of Wnt signaling by TNS4 and YAP signaling by TNS1; Abbreviations: low-density lipoprotein receptor–related protein (LPR), Frizzled (FZD), adenomatous polyposis coli (APC), axis inhibition protein 1 (AXIN), casein kinase 1 alpha (CK1α), glycogen synthase kinase 3 beta (GSK3β), lymphoid enhancer-binding factor (LEF), T cell factor (TCF), yes-associated protein (YAP).

Notably, TNS4 is implicated in canonical Wnt signaling (Figure 5). In the absence of the Wnt ligand, cytosolic β-catenin undergoes sequential phosphorylation by kinases CK1α and GSK3β within an AXIN/APC-scaffolded degradation complex. This targets β-catenin for ubiquitination and proteasomal degradation, maintaining low nuclear β-catenin levels [106]. Upon Wnt binding to coreceptors, β-catenin phosphorylation and degradation are inhibited. Consequently, β-catenin accumulates, translocates into the nucleus, and forms a complex with TCF/LEF transcription factors to activate Wnt target genes [106]. TNS4 was found to be highly expressed in colon cancer samples and cell lines, including SW480, SW620, and HT29 [107]. Intriguingly, TNS4 interacted with β-catenin specifically in the nucleus, despite the presence of both proteins in the cytosol [107]. TNS4 knockdown suppressed colony formation capacity, anchorage-independent growth, and cell invasion to levels similar to β-catenin knockdown in SW480 cells [107], suggesting that TNS4 modulates the tumorigenic potential of colon cancer cells via the β-catenin-dependent pathway, although the precise mechanisms remain unclear. Further supporting this link, β-catenin inhibition reduces TNS4 expression in HCT116 cells, while β-catenin overexpression increases it [108]. This indicates that TNS4 is upregulated by aberrant Wnt signaling. Thus, hyperactivated Wnt signaling contributes to tumorigenesis, with TNS4 being upregulated and integrated into Wnt signaling to exert its oncogenic role in colorectal cancer.

Yes-associated protein (YAP) functions as a nuclear transcriptional co-activator, regulating gene expression and proliferation by binding both the TEA domain (TEAD) transcription factors of the Hippo pathway and β-catenin in the Wnt pathway [109]. When Wnt signaling is inactive, cytoplasmic YAP becomes associated with β-catenin within the destruction complex. Upon Wnt pathway activation, YAP dissociates from the destruction complex and migrates to the nucleus to activate the Hippo pathway [110]. The activated Hippo pathway phosphorylates YAP, which then inhibits canonical Wnt signaling by either inhibiting nuclear accumulation of β-catenin or sequestering Src homology 2 domain-containing tyrosine phosphatase (SHP2) in the cytoplasm, thereby preventing SHP2 from promoting β-catenin-mediated transcription. YAP also acts as a downstream effector within the non-canonical Wnt signaling axis [109].

In an HCC mouse model, elevated tissue viscoelasticity induced TNS1 expression and activated integrin β1, RhoA, and YAP, collectively driving HCC progression [111]. Depletion of TNS1 or integrin β1 or disruption of their interaction via TNS1 mutation suppressed proliferation and YAP activity [111]. Consistently, pharmacological inhibition of ROCK-GTPase and myosin 2 reduced cellular contractility, as evidenced by increased circularity and decreased proliferation and YAP target gene expression. These findings define a TNS1–integrin β1–RhoA–YAP mechanotransduction axis that promotes HCC proliferation and invasion (Figure 5) [111]. Although YAP primarily activated Hippo signaling in this model, TNS1 may indirectly become involved in Wnt pathways through YAP regulation, which warrants further validation.

### 4.5. Tensins, Fibrillar Adhesion Formation, and AMPK

Integrin-based adhesion complexes, including focal adhesions and elongated fibrillar adhesions, primarily mediate cell–matrix adhesion across most cell types [39,42]. TNS3 predominantly localizes to fibrillar adhesions, while TNS1 is present in both focal and fibrillar adhesions [44]. TNS1 and TNS3 play critical, non-redundant roles in fibrillar adhesion formation. Silencing TNS1 or TNS3 in AMPK-deficient fibroblasts led to reduced fibrillar adhesion formation and fibronectin fibrillogenesis [43]. Furthermore, in human lung fibroblasts treated with TGF-β, TNS1 knockdown decreased the number and length of fibrillar adhesions [112].

Fibrillar adhesions derive from focal adhesions. During focal adhesion assembly, activated integrins link cells to the ECM. However, they cannot directly bind actin to transmit mechanical force and drive cell motility. Tensins bridge this gap: their ABDs bind actin, while their PTB domains bind integrin cytoplasmic tails. Importantly, TNS1 and TNS3 contribute to integrin activation, which is dependent on this tensin–integrin interaction, as evidenced by the finding that overexpression of TNS1 or TNS3 mutants disrupting this interaction failed to enhance integrin activity in telomerase-immortalized fibroblasts lacking endogenous tensins [43]. Moreover, overexpression of GFP-TNS3 in talin1-deficient cells (talin activates integrins and links them to actin) produced only a modest increase in integrin activity, indicating that tensin’s ability to enhance integrin activity also depends on talin. Based on these findings, Georgiadou et al. propose the talin–tensin switch hypothesis: talin1 initially triggers integrin activity within focal adhesions, and tensins subsequently sustain it in fibrillar adhesions, where talin is largely absent [43]. Although this hypothesis warrants further exploration, it strongly underscores the essential role of tensins in integrin activation.

**Figure 6:**
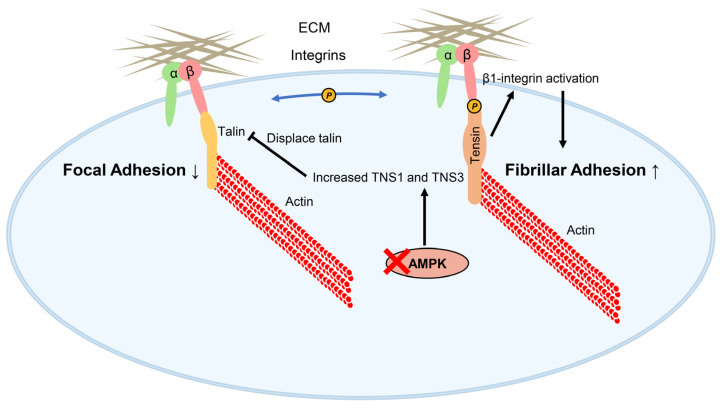
Mechanisms of AMPK-regulated fibrillar adhesion formation through TNS1 and TNS3. The cross refers to deficiency of AMPK, “*p*” refers to phosphorylation of proteins. The blue arrow indicates the switch between phosphorylated and unphosphorylated state, black arrows point to the results of the events, “↑/↓” refer to up/down-regulation.

The expression and function of tensins are modulated by AMPK, a serine/threonine kinase and key metabolic sensor that is crucial for maintaining energy homeostasis by regulating processes like lipogenesis, glycolysis, the tricarboxylic acid cycle, cell cycle progression, and mitochondrial dynamics [113]. AMPK knockout in mouse embryonic fibroblasts or silencing in telomerase-immortalized fibroblasts increased the expression of TNS3 or TNS1, respectively, concurrently enhancing tensin-mediated β1-integrin activation and fibrillar adhesion formation (Figure 6) [43]. These findings demonstrate that tensins act as signal transducers, functionally linking cellular energy sensing via AMPK to the regulation of cell adhesion and migration.

## 5. Regulatory Roles and Clinical Relevance of Tensins in Cancer

The signaling crosstalk orchestrated by tensins manifests as distinct pathological outcomes across cancer types. While TNS1–3 exhibit context-dependent dual roles as tumor suppressors or oncoproteins, TNS4 predominantly functions as an oncogenic driver. This section delineates the clinical evidence and molecular mechanisms underlying their cancer-specific and context-dependent contributions.

### 5.1. TNS1: Anti-Tumorigenic Roles in Prostate Cancer with Context-Dependent Oncogenicity

#### 5.1.1. Anti-Tumorigenic Roles in Prostate Cancer

TNS1 plays an anti-tumorigenic role in prostate cancers (Table 1). Specifically, in DU145 and PC3 prostate cancer cells, overexpression of the transcription factor hepatic leukemia factor (HLF) induced TNS1 transcription, subsequently activating the p53 pathway and reducing proliferation, migration, and invasion [114]. Importantly, depleting TNS1 reversed HLF’s anti-tumor effects both in vitro and in vivo, inhibiting tumor growth and metastasis [114]. However, clinical validation through human tissue studies is needed to confirm these findings.

#### 5.1.2. Oncogenic Roles in Colorectal, Liver, Gastric Cancers, and Leukemia

Conversely, TNS1 exerts oncogenic effects in colorectal, gastric, liver cancers, and leukemia (Table 1).

In colorectal cancer (CRC), multiple studies consistently support TNS1’s oncogenic function. Zhou et al. confirmed elevated mRNA/protein expression using Oncomine and Human Protein Atlas (HPA) data, and TCGA-based survival analysis linked high TNS1 mRNA levels to poor survival [70]. This trend was reinforced by Zhang et al.’s Kaplan–Meier analysis of 362 CRC patients, where low TNS1 expression was associated with improved overall survival and disease-free survival [115]. Further supporting this, Mi et al. predicted TNS1 as a target of miR-31-5p, with high TNS1 expression correlating with poor survival in The Cancer Genome Atlas (TCGA) and The University of ALabama at Birmingham CANcer data analysis Portal (UALCAN) databases [49]. Functionally, TNS1 knockdown in SW620 cells decreased proliferation and invasion [70], though the precise mechanisms require further investigation.

In HCC, increased viscoelasticity induced TNS1 expression alongside integrin β1, RhoA, and YAP activation, promoting progression [111]. Notably, knocking down TNS1 or integrin β1, or mutating TNS1 to impair integrin β1 binding, reduced proliferation and active YAP [111]. Consistent with this, pharmacological inhibition of ROCK-GTPase and myosin 2 increased cell circularity, lowered proliferation, and reduced YAP target gene expression, defining a TNS1–integrin β1–RhoA–YAP mechanotransduction axis [111]. Similarly, in gastric cancer (GC), TNS1 expression was elevated in peritoneal metastases compared to primary tumors, and its knockdown significantly reduced cell proliferation [116].

Beside solid tumors, TNS1 also promotes growth of leukemia cells. In the U937 and HL60 acute myeloid leukemia cell lines, it activates the PI3K/Akt/mTOR signaling pathway, and its knockdown suppresses proliferation [71].

#### 5.1.3. Dual Roles in Bladder, Lung, and Breast Cancers

TNS1 also exhibits context-dependent roles in bladder, lung, and breast cancers (Table 1).

In bladder cancer (BCa), the lncRNA MAGI2-AS3 targets miR-31-5p to promote TNS1 expression in T24 and J82 cells, inhibiting cell migration and invasion [50], suggesting a tumor-suppressive role. Clinically, Duan et al. analyzed TCGA RNA-seq data (414 tumors vs. 19 normal samples) and observed significant TNS1 downregulation in tumors. Subsequent examination of 45 paired clinical samples confirmed markedly reduced TNS1 expression in advanced-stage tumors (T3-4 vs. T1-2), with chi-square tests demonstrating associations between low expression and lymph node metastasis as well as poor prognosis [47]. Conversely, Luo et al. integrated TCGA and GSE13507 data and reported that reduced TNS1 expression correlated with negative lymph node metastasis, while high expression predicted poorer overall survival [117], indicating oncogenic potential. Furthermore, Zhang et al. documented elevated TNS1 prevalence in deceased muscle-invasive bladder cancer (MIBC) patients compared to survivors [121], implying subtype-specific oncogenicity. These contradictory clinical findings may result from methodological variations: although both studies utilized TCGA data and paired samples, Duan et al. analyzed the TCGA cohort (414 tumors/19 normal samples) and 45 paired samples separately, whereas Luo et al. combined both cohorts. Divergent thresholds for defining “high” versus “low” expression and unaccounted for tumor heterogeneity—where samples potentially represented distinct molecular subtypes not distinguished in either study—could further explain the discordant results.

In lung cancer, conflicting reports also exist. Duan et al. analyzed 36 paired NSCLC samples and found significantly higher TNS1 mRNA expression in tumor tissue, correlating with poor prognosis [47]. Mechanistically, TNS1 overexpression in A549 and H460 cells increased p-AKT/AKT and p-mTOR/mTOR ratios and RhoA activity. Critically, Akt or RhoA inhibitors blocked TNS1-induced growth, supporting oncogenicity via the Akt/mTOR/RhoA axis [47]. Similarly, ZNRD1-AS1 overexpression upregulated TNS1 in H1299 cells, promoting proliferation/migration [51]. In contrast, Liu et al. injected H1915 and A549 cells into the left ventricle of nude mice, collected brain metastases, and found upregulated microRNA-522-3p, which enhanced brain metastasis in vivo by suppressing TNS1 [118]. Likewise, Zhu et al. reported that miR-31-5p promoted A549 cell proliferation and migration in vitro and tumor growth in vivo by inhibiting the TNS1/p53 axis [119], highlighting tumor-suppressive roles.

In breast cancer, functional dichotomy is evident despite limited clinical data. The lncRNA MaTAR25 upregulates TNS1 in 4T1 triple-negative cells [120]. Its knockout downregulated TNS1, causing actin reorganization, reduced focal adhesions/microvilli, and suppressed proliferation/migration/invasion in vitro and tumor growth/metastasis in vivo [120], indicating pro-tumorigenicity. Conversely, miR-548j inhibits TNS1 and activates Cdc42, promoting invasion/metastasis [48].

These contradictions may stem from cellular heterogeneity. For example, TNS1 promotes cell migration in 4T1 breast cancer cells [120] but inhibits invasion of MCF-7, SKBR3, and MDA-MB-231 cells [48]. Strikingly, opposing effects occur even within the same cell line: Duan et al. reported pro-growth effects [47], whereas Liu et al. and Zhu et al. observed suppression in A549 cells [118,119]. Notably, methodological variations exist: Duan et al. assessed cell growth using MTT assays [47], whereas Liu et al. employed colony formation assays [118]. Additionally, Liu et al. and Zhu et al. included in vivo experiments [118,119], while Duan et al. did not [47]. These methodological differences may also contribute to the observed discrepancies.

### 5.2. TNS2: Tumor-Suppressive Dominance with Isoform-Specific Oncogenic Exceptions

Compared to TNS1, evidence elucidating TNS2 functions in cancer remains relatively limited. Bioinformatic analyses of public databases indicate consistent TNS2 downregulation across head and neck, esophageal, breast, lung, liver, and colon carcinomas [13]. Notably, low TNS2 expression correlated with poor overall prognosis in breast, lung, and bladder cancer cohorts and predicted inferior relapse-free survival in specific datasets [13], suggesting tumor-suppressive roles. Mechanistically, silencing TNS2 in HeLa and A549 cells elevated phosphorylation of IRS1, Akt, Mek, and Erk, accompanied by increased IRS1 protein levels, consequently promoting proliferation and colony formation. These findings indicate that TNS2 inhibits proliferation via suppressing the PI3K/Akt/mTOR and MAPK/Mek/Erk pathways [13]. Further supporting this tumor-suppressive function, high TNS2 expression in human liver tissue inhibits proliferation and induces apoptosis in HCC cell lines through direct DLC1 interaction [34].

However, context-dependent oncogenic roles exist. Specifically, TNS2 isoform 3 (a shorter variant) was overexpressed in 46% of HCC tumor samples versus adjacent tissue, with significant correlations to venous invasion, tumor microsatellite formation, and tumor nanoencapsulation [122], indicating an isoform-specific oncogenic function. In pancreatic cancer, Cheng et al. revealed concurrent TNS2 and AXL upregulation in pancreatic ductal adenocarcinoma (PDAC) through tumor tissue microarray analysis of 33 patients, supported by in vitro evidence [123]. This association was further confirmed by Zhu et al.’s large-scale study of 8280 cases and 6728 controls [124], implicating TNS2 in pancreatic tumorigenesis.

### 5.3. TNS3: Context-Specific Tumor Suppression Versus ESCC Oncogenicity

#### 5.3.1. Anti-Tumorigenic Roles in Lung, Glioblastoma, and Kidney Cancers

Functions of TNS3 in cancer exhibit cancer type and cell context specificities. Evidence supports tumor-suppressive roles in lung, glioblastoma, and kidney cancers through distinct mechanisms (Table 2). TCGA data show miR-200a-3p upregulation in LUSC and LUAD tumors [125]. In A549 cells, miR-200a-3p promoted cell migration by directly suppressing TNS3 expression [125], indicating TNS3’s anti-tumorigenic function in lung cancer. In glioblastoma, MSI1 directly interacts with the TNS3 mRNA 3’UTR to inhibit its translation, thereby activating RhoA and promoting migration [53].

In RCC, comparative analysis of 223 tumors and 48 normal kidney cortex tissues revealed significantly reduced TNS3 mRNA in tumor samples, where a higher tumor grade correlated with lower expression [52]. Consistently, immunohistochemistry (IHC) shows TNS3 in normal renal tubules but reduced/absent expression in 41% of RCC tumors [52]. This downregulation correlates with TNS3 promoter hypermethylation in RCC [56], indicating epigenetic silencing mechanisms. Functionally, TNS3 inhibits migration/invasion in HEK293 cells without affecting proliferation [52], while the histone methyltransferase MLL3 promotes TNS3 expression and suppresses migration in 769-P cells [57], though the precise mechanisms remain unclear.

#### 5.3.2. Oncogenic Role in Esophageal Cancer

TNS3 exhibits a clear oncogenic role in ESCC, showing high expression that correlates with malignancy and poor prognosis (Table 2) [73]. Correspondingly, knocking down TNS3 significantly inhibited ESCC cell proliferation both in vitro and in vivo [73]. Furthermore, the oncogenic circular RNA hsa_circ_0001165, mediated by EIF4A3, facilitates ESCC progression by upregulating TNS3 via the miR-381-3p/TNS3 pathway [126]. Silencing hsa_circ_0001165 reduced proliferation, migration, and invasion, and the effects were counteracted by TNS3 overexpression [126].

#### 5.3.3. Dual Roles in Thyroid, Gastric, and Breast Cancers

TNS3 exhibits context-dependent roles in thyroid, gastric, and breast cancers (Table 2). TNS3 was observed with high mRNA levels in normal thyroid tissues but low levels in most thyroid carcinomas and non-functioning thyroid follicular adenomas [54,55], suggesting a tumor-suppressive role in thyroid cancer. Conversely, however, high TNS3 expression was strongly associated with lymph node metastasis and poor survival in papillary thyroid carcinoma (PTC) [127], indicating functional variations across subtypes. In gastric cancer, TNS3 exhibits differential effects on tumorigenesis versus metastasis. An analysis of samples from 90 patients revealed more frequent TNS3 protein detection in moderately differentiated tumors compared to poorly/non-differentiated types [128], implying a potential promotion of tumorigenesis rather than metastasis. As a scaffold protein crosslinking diverse signaling pathways, TNS3 may exert distinct influences on proliferative and migratory pathways, potentially explaining these divergent outcomes.

**Table 2:** Clinical and mechanistic profiles of TNS3 in different cancers.

Cancer	Effects	Clinical Correlation (Sample)	Molecular Mechanisms
Renal cell carcinoma	↓	↓mRNA/protein = ↑tumor grade (223 tumors vs. 48 normal samples) [52]	Unclear
Esophageal squamous cell carcinoma	↑	↑protein = ↓OS (153 paired samples) [73].	Unclear
Thyroid cancer	↓	↓mRNA in most tumors (28 normal samples vs. 45 tumors) [54]	Unclear
	↓	↓mRNA in non-functioning thyroid follicular adenomas (18 normal samples vs. 50 tumors) [55]	
Papillary thyroid carcinoma	↑	↑mRNA = lymph node metastasis, ↓OS (GSE29265, GSE33630, GSE3467, GSE3678, GSE58545) [127]	
Breast cancer	↓	↓protein in poorly differentiated tumors [128]	Activating DLC1 in MCF10A cells [16]
	↑		Inhibiting DLC1 in MDA-MB-231 and MDA-MB-468 cells [58,129]

Notes: In the “Effects” column, “↑/↓”refers to oncogenic/tumor-suppressive; in the “Clinical Correlation (Sample)” column, “↑/↓” refers to up/down-regulation.

The roles of TNS3 in breast cancer are notably context-dependent. For instance, EGF treatment downregulates TNS3 while upregulating TNS4 in MCF10A cells [26], where TNS3 inhibits migration and metastasis through DLC1 interaction and RhoA inactivation [16]. Importantly, this TNS3–TNS4 switch is cell context-specific, as it does not occur in colorectal cancer cells where EGF upregulates both [81]. Conversely, exogenous introduction of SUV420H2 into MDA-MB-231 breast cancer cells downregulated TNS3 and reduced invasion, suggesting that TNS3 can facilitate migration/invasion [58]. Consistently, TNS3 knockdown inhibited cell migration in MDA-MB-468 breast cancer cells, though interestingly in an EGF induction-dependent manner [129]. TNS3 also exhibits contrasting effects on tumorigenesis versus metastasis. Analysis of TNS1–3 expression in 90 gastric cancer patients revealed that the TNS3 protein was more frequent in moderately differentiated tumors compared to poorly/non-differentiated types [128], suggesting a potential role in promoting tumorigenesis rather than metastasis. Furthermore, constitutive miR-375 expression in breast cancer cells directly targets TNS3, enhancing tumor-associated macrophage migration and facilitating tumorigenesis [130], highlighting TNS3’s role in modulating the tumor microenvironment beyond direct effects on cancer cells.

These conflicting functional outcomes in MCF10A, MDA-MB-231, and MDA-MB-468 cells partly stem from cell-specific DLC1 status. Although all these cell lines are DLC1-positive, MCF10A cells exhibit very low CDK5 activity with autoinhibited DLC1 [100], enabling TNS3 to activate DLC1 and inhibit RhoA signaling. In contrast, MDA-MB-231 and MDA-MB-468 cells harbor active DLC1, where TNS3 instead inhibits DLC1 to activate RhoA and promote migration. EGF treatment further modulates TNS3 function by facilitating TNS3–EGFR interaction [10] and activating Src-mediated phosphorylation of the TNS3 SH2 domain [10,32]. This phosphorylation is essential for efficient binding of ligands such as p130Cas and contributes critically to TNS3’s biological activity [32]. Consequently, mutations in EGFR or Src across different cellular contexts may differentially redirect TNS3’s functional impact on tumor progression.

### 5.4. TNS4: Pan-Cancer Oncogenic Driver with Prostate-Specific Tumor Suppression

#### 5.4.1. Predominant Oncogenic Roles Across Various Cancers

Numerous clinical studies collectively demonstrated TNS4’s predominant oncogenic functions across diverse cancers (Table 3). The most compelling oncogenic evidence emerges in gastric cancer. qRT-PCR detection of 114 paired samples confirmed TNS4 mRNA upregulation in tumors, correlating with poor differentiation, serosal invasion, nodal metastasis, and diminished survival [131]. Consistent with this, an analysis of 134 stage II/III GC patients receiving S-1 adjuvant chemotherapy associated higher TNS4 expression with poorer 5-year survival [132], while IHC of 80 cases linked upregulated protein levels to disease progression and poor prognosis [133]. This upregulation is partly explained by hypomethylation of its promoter region in gastric cancer cell lines, which correlates with enhanced proliferation and migration [134]. Regarding cancer metastasis, RNA-seq data of GC/lymph node tissues revealed metastatic lymph node-specific TNS4 upregulation, validated by qPCR/Western blot [135]. Although contrasting observations exist, for example, an IHC assessment of 89 GC patients identified TNS4 positivity (55.06%) primarily in moderately differentiated intestinal-type tumors without mucinous components (a subtype associated with better prognosis), it should be noticed that no survival correlation was observed [136]. Despite robust clinical associations, mechanistic insights into gastric cancer remain limited.

Transitioning to esophageal cancer, TCGA data revealed TNS4 mRNA upregulation in ESCC tumors [89]. Furthermore, IHC of 134 samples showed 50.7% TNS4 positivity in tumors versus only 6.7% in adjacent tissues, with positive expression significantly correlating with lymph node metastasis and reduced overall survival [89]. Similarly in head and neck cancers, integrated analysis of TCGA and independent cohorts demonstrated progressive TNS4 upregulation from normal to dysplastic to tumor tissues in head and neck squamous cell carcinoma (HNSCC), where high expression predicted poorer survival, which was validated by significant protein overexpression in 12 paired tumor/normal samples [137]. Although clinical data support oncogenicity in these cancers, underlying mechanisms require further elucidation.

In contrast, colorectal cancer exhibits well-defined oncogenic mechanisms alongside clinical evidence. Database analyses (UALCAN/GEPIA) reveal significant TNS4 mRNA upregulation in tumor tissues, a finding corroborated by an immunohistochemical assessment of 92 paired paraffin blocks showing elevated protein expression, particularly in late-stage (III-IV) versus early-stage (I-II) disease [138]. Functionally, TNS4 enhances oncogenic growth in LS174T, DLD1, WiDr, and DiFi CRC cell lines [82] and promotes ECM invasion in 3D models while suppressing proliferation [139]. Notably, TGF-β1 stimulation upregulated TNS4 and EMT markers, increasing migration/invasion in SW620 and HCT116 cells [140]. Additionally, TNS4 stabilizes Src post-transcriptionally, promoting EMT and potentially metastasis in SW620 and HCT116 cells [65]. Concurrently, it facilitates migration/invasion via β-catenin/c-Myc-mediated aerobic glycolysis in HCT116 and RKO cells [138]. Epigenetically, PRMT1-mediated H4R3me2a recruits SMARCA4 to enhance EGFR signaling and TNS4 expression [84], while a SMARCA4R mutant augments SWI/SNF activity to reinforce EGFR/TNS4 transcription and proliferation [85]. Collectively, these findings establish TNS4 as a multi-mechanism oncoprotein in CRC, involving TGF-β, Src stabilization, and Wnt and EGFR signaling.

In NSCLC, TCGA data associate elevated TNS4 with poor overall/disease-free survival and advanced stages, confirmed by tissue microarrays (20 LUAD vs. 14 normal samples) [141]. Further supporting this, a meta-analysis of seven datasets links high TNS4 expression to positive lymph node metastasis, larger tumors, and reduced survival [142]. Mechanistically, EGF upregulates TNS4 through activating STAT3, mediating the invasion of H125 and A549 cells [143], and TNS4 overexpression in A549 and NCI-H1299 LUAD cells activates TGF-β1 expression, inducing EMT [67]. Furthermore, this pro-tumorigenic role extends to gallbladder cancer, where GPRC5A promotes metastasis via the JAK2-STAT3/TNS4 axis [144], though clinical evidence is absent.

**Table 3:** Clinical and mechanistic profiles of TNS4 in different cancers.

Cancer	Effects	Clinical Correlation (Sample)	Molecular Mechanisms
Gastric cancer	↑	↑mRNA/protein = ↑metastasis, ↓OS (114 paired samples) [131]	unclear
	↑	↑mRNA = ↓OS (134 patients) [132]	
	↑	↑mRNA/protein = ↓survival (80 paired samples) [133]	
	↑	↑mRNA/protein = ↑lymph node metastasis (7 paired samples) [135]	
	↓	↓protein = ↓differentiation (89 tumors vs. 20 normal samples) [136]	
Esophageal squamous cell carcinoma	↑	↑mRNA/protein = ↑metastasis, ↓OS (TCGA data and 134 paired samples) [89]	unclear
Head and neck squamous cell carcinoma	↑	↑mRNA/protein = ↓OS (patients from Stomatological Hospital at Nanjing Medical University, TCGA, GSE37991, GSE58911, GSE83519, GSE25099, GSE55550 and GSE30784) [137]	unclear
Colorectal cancer	↑	↑mRNA/protein = later stage (UALCAN and GEPIA databases + 92 pairs of CRC tissues) [138]	TGF-β1 upregulating TNS4 [140]
			TNS4 stabilizing Src post-transcriptionally [65]
			promoting β-catenin/c-Myc-mediated aerobic glycolysis [138]
			SMARCA4 enhancing EGFR signaling and TNS4 expression [84]
Non-small cell lung cancer	↑	↑protein = ↑metastasis, ↓OS/DFS (20 LUAD tumors vs. 14 controls) [141]	EGF/STAT3 upregulating TNS4 [143]
	↑	↑mRNA = ↓OS/PFS (TCGA-LUAD data) [142]	TNS4 activating TGF-β1, inducing EMT [67]
Gallbladder cancer	↑	Not available	GPRC5A/JAK2-STAT3/TNS4 axis [144]
Prostate cancer	↓	↓mRNA in tumors (4 pairs + 3 tumors) [11]	TNS4 inhibiting EGFR [90]
Breast cancer	↑	↑protein = ↑tumor size, grade, metastasis (1409 cases) [145]	STAT3 upregulating TNS4 [146]
	↓	↓mRNA on tumors (TCGA-BRCA data) [147]	TNS4 targeting VEGFA through c-Cbl-mediated β-catenin downregulation [147]

Notes: In the “Effects” column, “↑/↓”refers to oncogenic/tumor-suppressive; in the “Clinical Correlation (Sample)” column, “↑/↓” refers to up/down-regulation.

#### 5.4.2. Anti-Tumorigenic Role in Prostate Cancer

Despite its widespread oncogenicity, TNS4 exhibits an anti-tumorigenic function specifically in prostate cancer (Table 3). TNS4 expression is downregulated in prostate tumor tissues and cancer cell lines [11]. Mechanistically, ΔNp63α transcriptionally regulates TNS4 in normal prostate epithelial cells, modulating cell adhesion [45]. Downregulation of both TNS4 and ΔNp63 correlates with prostate cancer progression from primary to metastatic disease [45]. Furthermore, TNS4 is downregulated in paclitaxel-resistant PC-3 and DU145 prostate cancer cells, where its expression negatively correlates with EGFR levels, indicating that TNS4 exerts anti-tumor effects via EGFR inhibition [90].

Unlike other tensins, TNS4 displays selective expression restricted to only a few tissues, including prostate tissue [11,36,74,148]. High TNS4 expression is essential for normal prostate cell function, and its downregulation may promote malignant transformation. For instance, TNS4 is specifically expressed in prostate basal epithelial cells. TNS4 knockdown in nonmalignant RWPE-1 prostatic epithelial cells suppressed proliferation and induced G1/S cell cycle arrest, suggesting a role in maintaining the basal compartment [74]. Additionally, TNS4 is cleaved by caspase-3 during apoptosis of normal prostate epithelial cells, and the resultant fragments can further promote apoptosis [148]. TNS4 downregulation may impair this pro-apoptotic function, facilitating abnormal proliferation. Moreover, TNS4 negatively regulates EGFR expression in prostate cancer cells [90], contrasting with findings in other cancers. Although the basis for this tissue-specific difference remains unknown, EGFR inhibition likely mediates TNS4’s anti-tumorigenic function in prostate cancer.

#### 5.4.3. Dual Roles in Breast Cancer

TNS4 exhibits dual functions in breast cancer (Table 3). In MCF10A cells, EGF stimulation upregulates TNS4 and promotes migration [26]. Furthermore, immunohistochemical analysis of 272 invasive breast carcinomas revealed elevated TNS4 protein expression correlating with a high tumor grade and lymph node metastasis [26], a finding confirmed in a larger tissue microarray study (n = 1409) [145]. Notably, increased TNS4 is associated with upregulated p-Akt, PI3K, and N-cadherin, implicating it in FAK-PI3K-Akt signaling [145]. Mechanistically, constitutively active Signal Transducer and Activator of Transcription 3 (STAT3) upregulates TNS4 in both mouse model and human MCF10A cells, enhancing cell migration and invasion [146]. Collectively, these studies indicate that TNS4 promotes breast cancer progression.

Paradoxically, TCGA and CPTAC databases show downregulated TNS4 mRNA across breast cancer subtypes (luminal, HER2+, triple-negative) [147]. Functionally, TNS4 inhibits proliferation, migration, and angiogenesis in MCF7 cells and suppresses tumor growth in nude mice by targeting VEGFA through c-Cbl-mediated β-catenin downregulation [147].

An apparent contradiction emerges: high TNS4 protein positivity in tumors [26,145] versus low mRNA levels [147]. Although based on different sets of samples, this contradiction suggests significant post-transcriptional regulation. Future studies should concurrently examine both mRNA and protein expression. Furthermore, the opposing functional outcomes in MCF10A (pro-migratory) and MCF7 (anti-migratory) cells further indicate that TNS4’s role may be subtype-dependent, underscoring the need for subtype-specific investigations in breast cancer.

### 5.5. Somatic Mutations in Tensins: Limited Prevalence and Uncertain Significance

In contrast to the well-established roles of tensin expression dysregulation, somatic alterations in *TNS1–4* genes through mutations represent rare events with undefined clinical significance. Analysis of large-scale genomic datasets (e.g., TCGA PanCancer Atlas) reveals mutation frequencies typically below 3% across most malignancies, often within background mutation rates. The limited literature primarily documents three categories of alterations: gene amplifications, fusions, and sporadic point mutations.

*TNS1* amplification has been reported in sporadic colorectal carcinomas (53 cases) [149], while co-amplification of TNS4 within the 17q21.2 locus, which encompasses GJC1, IGFBP4, TNS4, and TOP2A, was observed in ER-positive/HER2-amplified breast cancers (70 cases), suggesting potential oncogenic cooperativity [150]. Oncogenic fusions involving tensins include *TNS1*::*BRAF* in BRAF-negative papillary thyroid carcinomas (identified in a cohort of 62 cases) [151] and *FGFR2*::*TNS1* detected in circulating tumor DNA from biliary tract cancer patients (102 cases) [152]. Point mutations remain exceptionally scarce, with isolated reports including *TNS1* variants in peripheral T-cell lymphoma (1/26 patients) [153], a *TNS1* p.S1309Y missense mutation associated with enhanced migration in clear cell renal cell carcinoma [154], and a splice-site mutation of TNS1 (NM_022648.7:c.2999-1G > C) identified in a synchronous multiple myeloma/thyroid carcinoma patient [155].

Critically, these alterations lack experimental validation and clinical correlations, positioning them as minor contributors to cancer pathogenesis. The predominant oncogenic mechanisms involving tensins remain rooted in dysregulated expression and signaling modulation.

## 6. Biomarker and Therapeutic Target Potential of Tensins in Cancer

Emerging evidence collectively demonstrates tensins’ critical involvement in cancer progression, prompting an exploration of their clinical translational potential. Advances in high-throughput sequencing and bioinformatics applied to large clinical cohorts facilitate the substantiation of tensins’ utility as diagnostic and prognostic biomarkers. Notably, TNS4’s predominant oncogenicity across diverse cancers highlights its therapeutic targeting potential, though this requires further preclinical and clinical validation.

### 6.1. Diagnostic Biomarker Potential

TNS2 exhibits notable diagnostic specificity for gastrointestinal stromal tumors (GISTs). Comparative analysis of 148 GISTs versus other sarcomas and gastrointestinal primary tumors revealed markedly elevated TNS2 expression in GISTs, with 71.4% showing intermediate/strong staining compared to only 2.9% in other sarcomas [156]. This differential expression addresses critical diagnostic challenges in distinguishing gastric GISTs from morphologically similar sarcomas that may arise in the stomach, including liposarcomas, leiomyosarcomas, and unclassified sarcomas [157]. Although the established markers KIT and DOG1 exhibit high sensitivity for GIST diagnosis (if at least one marker is positive [158]), their expression in other gastric sarcomas limits diagnostic specificity [159]. In the 148 analyzed tumors, which were all TNS2-positive, 147 cases (99.3%) were positive for at least one of the markers DOG1 and KIT, confirming good sensitivity. Although lacking prognostic correlation [156], TNS2 could be a valuable adjunct diagnostic marker for discriminating GISTs from other gastric sarcomas.

In oral squamous cell carcinoma, TNS3 demonstrates tissue-specific diagnostic value. An analysis of 46 paired tumor/non-cancerous tissues identified significant TNS3-203 transcript overexpression in malignant tissues [160].

### 6.2. Prognostic Biomarker Potential

#### 6.2.1. TNS1: Multi-Cancer Prognostic Utility via Modeling Strategies

*TNS1* demonstrates validated prognostic utility in bladder, colorectal, and gastric cancers, where its incorporation into multi-gene signatures predicts survival outcomes (Table 4). For MIBC prognosis, Zhang et al. divided TCGA-BLCA samples equally into training and validation sets. Through univariable Cox regression, they identified *KLK6*, *TNS1*, and *TRIM56* as optimal prognostic genes, with *KLK6*/*TNS1* overexpression linked to poorer survival [121]. They then developed a multivariable Cox model stratifying patients by risk scores, which revealed significant survival differences. This signature’s robustness was subsequently confirmed in both the TCGA validation set and an independent E-MTAB-1803 cohort [121].

Turning to CRC, Chen et al. evaluated autophagy and stroma status using GSE39582 microarray data. Their LASSO model selected *TNS1*, *TAGLN*, and *SFRP4* for a risk signature that emerged as an independent prognostic factor, later validated across GSE17538, GSE38832, and TCGA datasets and clinical samples from the First Hospital of China Medical University [161]. Furthermore, Liu et al. identified *TNS1* among five stroma-related genes [162]. Higher stromal scores based on these genes predicted poorer survival in 524 CRC patients, and this was validated in two independent cohorts [162].

In GC, Jiang et al. divided 221 GC samples into training/validation sets. Using LASSO Logistics analysis, they identified four genes (*COL14A1*, *TNS1*, *NUSAP1*, *YWHAE*) linked to peritoneal metastasis (PM) occurrence, where high *COL14A1*/*TNS1* expression increased PM risk [116]. The resulting risk score stratified patients into groups, with high-risk patients showing shorter overall and disease-free survival in both the training and validation sets [116]. The predictive accuracy was demonstrated by ROC analysis, where values of 0.811 in the training set and 0.786 in the validation set indicated good discriminative capacity for PM risk prediction. Notably, when integrated with TNM staging, predictive performance substantially improved to AUCs of 0.896 (training) and 0.884 (validation), approaching the threshold (0.9) for excellent discrimination [116]. The clinical relevance was further underscored in 36 PM versus 72 non-metastatic GC samples, where high risk scores predicted poorer survival [116].

#### 6.2.2. TNS3: Metastasis-Specific Biomarker in PTC

Meanwhile, in PTC, integrated bioinformatic analysis of five independent Gene Expression Omnibus datasets (GSE29265, GSE33630, GSE3467, GSE3678, GSE58545) demonstrated that TNS3 expression is strongly associated with lymph node metastasis [127]. Critically, elevated TNS3 expression predicted significantly reduced survival, confirming its value as a predictive biomarker for lymph node metastasis in PTC [127].

#### 6.2.3. TNS4: Core Component in Multi-Gene Signatures

*TNS4* demonstrates consistent prognostic value as an integral element in multi-gene signatures across malignancies, validated through progressively sophisticated bioinformatic methodologies (Table 5). In CRC, Song et al. established a radiation resistance signature (*LGR5*, *KCNN4*, *TNS4*, *CENPH*) through integrated analysis of sequencing data from radiation-resistant cells and TCGA cohorts [163]. This signature demonstrated significant correlation with radiotherapy outcomes in CRC patients, with AUCs of 1-, 3-, and 5-year curves reaching 0.87, 0.94, and 0.9, respectively, indicating reliable predictive ability [163].

For NSCLC, *TNS4* contributes to six distinct prognostic models reflecting methodological evolution. The initial work derived a seven-gene risk model from TCGA-LUAD/GDC data, demonstrating robust survival prediction (AUC > 0.8) validated through Kaplan–Meier/ROC analyses and GSE26939, with consistent performance across ages/TNM stages [164]. Subsequent studies refined this approach through multi-omics integration: Liu et al. developed a 14-gene hypoxia/ferroptosis signature via ssGSEA scoring of TCGA profiles refined by WGCNA and Cox-LASSO regression (AUC > 0.8) [165], while machine learning integration of GSE21656/GSE108214/TCGA data generated a novel three-gene predictor (*GPX8*, *BCAR3*, *TNS4*) [166]. Expanding this to inflammatory markers, Bao et al. constructed a six-gene LPS-related model using Cox proportional hazard regression coupled with LASSO penalization, where high risk scores predicted poorer prognosis as independent prognostic factors [167]. For metastasis-specific prediction, Yu et al. created an eight-gene lymph node metastasis signature through DEG/WGCNA analysis validated across GEO datasets (GSE68465, GSE42127, GSE50081) [168]. Most innovatively, Chen et al. pioneered a clinically translatable T-cell senescence model (*SLC2A1*, *TNS4*, *GGTLC1*) using scRNA-seq and T-cell assays, uniquely predicting immunotherapy response across eight GEO cohorts and clinical samples (AUC > 0.8) [169].

Remarkably, all *TNS4*-incorporated signatures demonstrated robust predictive capacity (AUC > 0.7) across diverse analytical frameworks, spanning conventional regression, machine learning, and single-cell technologies [164,165,166,167,168,169], with three signatures exceeding AUC 0.8 [164,165,169], indicating consistently strong prognostic performance. This versatility extends to pancreatic cancer, where integrated analysis of 50 TCGA-PDAC samples and 29 institutional patient profiles yielded a three-gene signature (*BCHE*, *ADH1A*, *TNS4*) via DEG screening and multivariate Cox regression. The model independently predicted adjuvant chemotherapy response while correlating with immune infiltration patterns and post-chemotherapy recurrence-free survival [170].

### 6.3. Therapeutic Target Potential

Tensins play significant roles in cancer-related signaling pathways and exhibit regulatory functions in malignancies, with earlier studies highlighting the predominant oncogenic activities of TNS4 across diverse cancers. This evidence signifies their potential as therapeutic targets. Although no drugs directly targeting tensins currently exist and clinical validation remains limited, insights from tensin-related proteins such as integrins offer valuable references.

Notably, Avastin (bevacizumab), a murine-derived monoclonal antibody against human integrin αvβ3 and vascular endothelial growth factor (VEGF), exerts antiangiogenic effects by blocking integrin–VEGFR interactions, thereby inhibiting angiogenesis induced by basic fibroblast growth factor (bFGF) and tumor necrosis factor-α (TNF-α) [171]. FDA-approved indications include first- or second-line metastatic breast cancer therapy, combination chemotherapy for metastatic colorectal cancer, and management of glioblastoma, metastatic renal cell carcinoma, and NSCLC [172]. Similarly, the cyclic pentapeptide cilengitide potently inhibits αvβ3/αvβ5 integrin-mediated adhesion and migration via suppression of the FAK/SRC/AKT pathway, inducing endothelial cell apoptosis. Preclinical in vitro studies confirm its significant antiangiogenic activity [173].

Such antibody- or peptide-based strategies could be adapted for TNS4 targeting. Given the minimal or absent TNS4 expression in most normal tissues, therapeutic interventions may minimize off-target effects. Furthermore, since TNS4 modulates activated EGFR levels [88,89] and acts as a downstream effector in the Ras/Raf/Mek/MAPK cascade [83], combinatorial targeting of TNS4 and EGFR represents a theoretically viable strategy for EGFR-dysregulated cancers. Challenges exist, however, as TNS4 participates in both oncogenic and tumor-suppressive pathways, complicating the balance between efficacy and toxicity. Consequently, while tensins hold theoretical therapeutic promise, further preclinical and clinical evidence is essential to substantiate their translational potential.

## 7. Discussion

The human tensin family members (TNS1, TNS2, TNS3, and TNS4) serve as critical scaffold proteins, regulating signaling crosstalk in fundamental cellular processes, including adhesion, migration, proliferation, and mechanotransduction. Clinical and mechanistic evidence consistently links their dysregulation to tumorigenesis and metastatic progression. All four tensins share evolutionarily conserved, tandemly arranged C-terminal SH2-PTB domains that mediate integrin binding, focal adhesion localization, and signal transduction functions. Crucially, structural divergence emerges in N-terminal domains: TNS1–3 contain ABDs enabling actin linkage and mechanical force transmission, whereas TNS4 lacks ABD and represents a distinct branch of the family specifically in mammals [36,38]. The absence of ABD shifts TNS4’s function toward signal amplification in focal adhesions, stabilizing oncogenic receptors like EGFR and promoting EMT and invasion [62,63,64,65,66,67,88,89], thus carrying out predominate oncogenic functions across carcinomas. Additional structural variations further differentiate between members: TNS2 uniquely possesses an N-terminal C1 domain [13], while TNS1 harbors a catalytically inactive PTP domain [18]. These architectural distinctions likely underpin functional diversification across cancer contexts.

The most striking feature of tensins, particularly TNS1 and TNS3, is their context-dependent duality, acting as either oncoproteins or tumor suppressors in different malignancies or even different subtypes of the same malignancy. A central question remains: what molecular determinants specify whether tensins function as oncoproteins or tumor suppressors? This represents the field’s primary challenge and requires further investigation to fully elucidate the underlying mechanisms. This paradox cannot be attributed solely to genetic mutation, though it is an important factor for some oncogenes or tumor suppressors like p53, because the mutation frequency of tensins remains low across most cancers. Instead, three interconnected factors (isoform variation, microenvironmental influences, and cell context specificity) likely determine functional outcomes.

Isoform-specific effects are exemplified by TNS2, where the short isoform 3 promotes HCC, contrasting with other isoforms [122]. Similar isoform-based duality likely exists for other tensins but remains unexplored in current studies, warranting focused investigation into isoform-specific functions. Microenvironmental factors also modulate tensins’ functions. In HCC, increased ECM viscoelasticity induces TNS1 and activates a stiffness-independent RhoA–YAP oncogenic axis [111]. This suggests the influence of the ECM’s physical properties on tensin behavior. Furthermore, constitutive miR-375 expression in breast cancer cells directly suppresses TNS3, enhancing tumor-associated macrophage migration and accelerating tumorigenesis [130]. This highlights TNS3’s role in modulating the tumor microenvironment beyond cancer cell-autonomous effects, where variable compositions of stromal cells may further impact tensin functionality. Nevertheless, our understanding of tensin’s roles across microenvironments remains limited and requires deeper investigation.

Perhaps the most significant determinants of tensin functionality reside within cancer cells themselves. Unlike transmembrane proteins, such as integrins, tensins mediate signaling through intracellular binding partners. Consequently, cell context specificity, defined by the differential availability of binding partners, dictates functional outcomes. This principle is vividly illustrated by TNS3’s opposing roles depending on DLC1 status: in DLC1-active cells like HFFs and H157/H1703 NSCLC cells, it inhibits DLC1 to promote RhoA-driven migration [15,44], whereas in DLC1-inactive MCF10A cells, it suppresses RhoA and inhibits motility [16,100]. Critically, binding partners exhibit interdependencies: when TNS1–PP1α interaction occurs, TNS1 binds and inhibits DLC1 to activate RhoA and promote migration, while weakening the TNS1–DLC1 interaction reduces migration [15,22]. Paradoxically, impairing the TNS1–PP1α interaction, which also weakens TNS1–DLC1 binding, leads to elevated migration [15,22]. Similarly, TNS4 exhibits contradictory effects on EGFR: it stabilizes EGFR in lung/bladder/esophageal cancers [88,89] but downregulates EGFR in prostate cancer cells [90]. This tissue-specific divergence may involve unidentified co-regulators, pending validation.

Crucially, the clinical interpretation of tensin functions faces additional complexity from methodological variations and tumor heterogeneity. In breast cancer, for example, discordant observations of high TNS4 protein positivity versus low mRNA levels [145,147] strongly suggest post-transcriptional regulation, emphasizing the necessity of multi-omics validation in biomarker studies. More notably, contradictory clinical correlations for TNS1 in bladder cancer, where Duan et al. reported low mRNA associated with lymph node metastasis [47] while Luo et al. linked high expression to poor prognosis [117], likely originate from analytical disparities, though both studies utilized TCGA data and paired samples. Specifically, Duan et al. separately analyzed the TCGA cohort (414 tumors/19 normal samples) and paired samples, whereas Luo et al. merged both datasets. Distinct molecular subtypes between cohorts and divergent thresholds for defining “high” versus “low” expression likely amplified these discrepancies.

Despite these challenges in functional interpretation, large-scale clinical analyses substantiate tensins’ diagnostic and prognostic biomarker utility. TNS2 shows promise as a diagnostic adjunct for discriminating GISTs from other gastric sarcomas [156], while TNS1 and TNS4 are increasingly incorporated into validated multi-gene signatures predicting survival and therapeutic response in bladder, colorectal, gastric, and lung cancers [50,121,161,163,164,165,166,167,168,169,170].

Notably, TNS4’s predominant oncogenicity across diverse cancers highlights its therapeutic targeting potential. Although direct TNS4-targeting drugs remain undeveloped currently and clinical evidence remains limited, established integrin-targeting modalities, such as antibody-/peptide-based strategies [171,172,173] could provide a reference for TNS4 targeting. Given its dual role as both an EGFR modulator [88,89] and downstream effector in Ras/Raf/Mek/MAPK signaling [83], combinatorial targeting of TNS4 and EGFR represents a rational strategy for EGFR-dysregulated cancers. The minimal or absent TNS4 expression in most normal tissues suggests favorable safety profiles. Challenges exist, as TNS4 participates in both oncogenic and tumor-suppressive pathways, complicating the balance between efficacy and toxicity. However, advances in artificial intelligence (AI) and machine learning (ML) provide opportunities to accelerate TNS4-targeted drug development. ML algorithms can systematically utilize multi-target networks to design synergistic therapies [174], facilitating the discovery of TNS4–EGFR dual inhibitors, while deep learning models predict binding affinities and optimize drug-like properties to reduce clinical attrition rates [175]. Integrating these approaches into network pharmacology frameworks [174] may provide a robust platform for overcoming TNS4’s functional complexity, accelerating the development of precision therapeutics with minimized off-target risks.

## 8. Conclusions and Future Perspectives

This review synthesizes current knowledge on the four human tensins (TNS1, TNS2, TNS3, and TNS4), including their structural basis, regulatory mechanisms, and clinical relevance. Their structural variation, notably the presence of ABDs in TNS1–3 versus ABD’s absence in TNS4, correlates with distinct cancer roles. TNS4 demonstrates predominant oncogenicity across carcinomas (except for prostate cancer), while TNS1 and TNS3 exhibit context-dependent duality as oncoproteins or tumor suppressors. Current evidence suggests that this functional switching is modulated by isoform specificity, microenvironmental cues, and dynamic binding partner availability. Critically, we consolidate tensins’ emerging diagnostic/prognostic utility—previously lacking systematic synthesis—as exemplified by TNS2 aiding GIST classification and TNS4 integration into multi-gene signatures.

Limitations should be contextualized within the current evidence landscape. First, this review focuses on cancer pathobiology, with normal physiological functions of tensins not being exhaustively addressed. Second, mechanistic ambiguities persist in understanding context-dependent functional switching; proposed determinants like tissue-specific co-regulators reflect plausible hypotheses derived from existing data, but their validation remains pending and represents a shared challenge for the field. Finally, TNS4’s therapeutic promise currently operates within a theoretical framework, constrained by inadequate clinical validation and the unresolved efficacy–toxicity balancing inherent to its pathway duality.

Looking forward, three priorities demand focused attention to propel tensin research. The first is deepening mechanistic insight into isoform-specific roles and microenvironmental regulation, particularly where preliminary evidence flags their significance (e.g., TNS2 isoform 3, the ECM–TNS1 axis) yet mechanistic understanding remains superficial. The second is adopting multi-omics frameworks with molecular subtyping rigor to resolve clinical inconsistencies, such as the TNS4 mRNA–protein discordance in breast cancer. The third is strategically leveraging computational tools like AI/ML-assisted network pharmacology to navigate TNS4’s signaling complexity and accelerate dual-targeting designs (e.g., TNS4–EGFR), though their utility requires empirical confirmation. Collectively, this unified framework could enable improved diagnostic/prognostic precision and inform therapeutic strategies for tensin-driven cancers as mechanistic understanding matures.

## Figures and Tables

**Table 1:** Clinical and mechanistic profiles of TNS1 in different cancers.

Cancer	Effects	Clinical Correlation (Sample)	Molecular Mechanisms
Prostate cancer	↓	Not available	Activating p53 signaling [114]
Colorectal cancer	↑	↑mRNA/protein = ↓OS (TCGA and HPA databases) [70]	Unclear
	↑	↑mRNA = ↓OS and DFS (362 patients) [115]	
	↑	↑mRNA = ↓survival (TCGA + UALCAN databases) [49]	
Hepatocellular carcinoma	↑	Not available	TNS1–integrin β1–RhoA–YAP mechanotransduction axis
Gastric cancer	↑	↑mRNA = ↑peritoneal metastasis (221 patients) [116]	Unclear
Acute myeloid leukemia	↑	Not available	Activating the PI3K/Akt/mTOR signaling [71]
Bladder cancer	↓	↓mRNA = ↑metastasis (TCGA data + 45 paired samples) [50]	Unclear
	↑	↑mRNA = ↓OS (TCGA data + GSE13507) [117]	
Non-small-cell lung cancer	↑	↑mRNA = ↓OS (36 paired samples) [47]	Activating Akt/mTOR/RhoA axis
	↓	Not available	microRNA-522-3p suppressing TNS1 [118]
	↓		miR-31-5p inhibiting TNS1/p53 axis [119]
Breast cancer	↓	Not available	MaTAR25 upregulating TNS1 [120]
	↑		miR-548j inhibiting TNS1 [48]

Notes: In the “Effects” column, “↑/↓”refers to oncogenic/tumor-suppressive; in the “Clinical Correlation (Sample)” column, “↑/↓” refers to up/down-regulation.

**Table 4:** Prognostic biomarker utility of *TNS1* in different cancers.

Cancer Type	Gene Signature	Prognosis	Validation Datasets
Muscle-invasive Bladder cancer	*KLK6*, *TNS1*, *TRIM56*	High risk scores predict poor OS	TCGA database and E-MTAB-1803 cohort [121]
Colorectal cancer	*TNS1*, *TAGLN*, *SFRP4*	High risk scores correlate with advanced tumors and poor OS	GSE39582 microarray, GSE17538, GSE38832, and TCGA datasets and clinical samples from the First Hospital of China Medical University [161]
	*ITGA7*, *PTPN14*, *SCG2*, *TNS1*, and *GRP*	High stromal scores correlate with advanced tumor stages and poor OS and relapse-free survival	GSE39582, GSE17536, and TCGA [162]
Gastric cancer	*COL14A1*, *TNS1*, *NUSAP1*, and *YWHAE*	High risk scores predict poor OS	221 samples from GSE62254 cohort [116]

**Table 5:** Prognostic biomarker utility of *TNS4* in different cancers.

Cancer Type	Gene Signature	Prognosis	Validation Datasets
Colorectal cancer	*LGR5*, *KCNN4*, *TNS4*, *CENPH*	High risk scores predict more radiation resistance, tumor progression, and poor PFS	TCGA database and GSE97543 [163]
Non-small-cell lung cancer	*NTSR1*, *RHOV*, *KLK8*, *TNS4*, *C1QTNF6*, *IVL* and *B4GALNT2*	High risk scores predict poor survival rate	TCGA-LUAD dataset and GSE26939 [164]
	*MAPK4*, *TNS4*, *WFDC2*, *FSTL3*, *ITGA2*, *KLK11*, *PHLDB2*, *VGLL3*, *SNX30*, *KCNQ3*, *SMAD9*, *ANGPTL4*, *LAMA3* and *STK32A*	High risk scores predict advanced tumor stage and poor survival rate	TCGA-LUAD and GSE31210 [165]
	*GPX8*, *BCAR3*, *TNS4*	High risk scores predict poor survival rate	GSE21656, GSE108214, and TCGA data [166]
	*VIPR1*, *NEK2*, *HMGA1*, *FERMT1*, *SLC7A* and *TNS4*	High risk scores predict poor survival rate	TCGA data and GSE37745 [167]
	*ANGPTL4*, *BARX2*, *GPR98*, *KRT6A*, *PTPRH*, *RGS20*, *TCN1* and *TNS4*	High risk scores predict poor OS	TCGA data, GSE68465, GSE42127, GSE50081 [168]
	*SLC2A1*, *TNS4*, *GGTLC1*	High risk scores predict poor OS and PFS	TCGA-LUSC, TCGA-LUAD, GSE19188, GSE30219, GSE37745, GSE50081, GSE29013, GSE31210, GSE4573, GSE68465 [169]
Pancreatic ductal adenocarcinoma	*COL14A1*, *TNS1*, *NUSAP1*, *YWHAE*	High risk scores predict better response to adjuvant chemotherapy after surgical resection and poor OS	TCGA data and 26 patients from Zhongshan Hospital of Fudan University [170]

## Data Availability

This review does not present new experimental data. For data availability, please refer to the respective original papers.

## Abbreviations

The following abbreviations are used in this manuscript:

ABD	Actin-binding domain
Akt	Protein kinase B
AML	Acute myeloid leukemia
AMPK	AMP-activated protein kinase
APC	Adenomatous polyposis coli
AXIN	Axis inhibition protein 1
BCa	Bladder cancer
BRCA	Breast cancer
CAF	Cancer-associated fibroblast
CK1α	Casein kinase 1 alpha
CRC	Colorectal cancer
DFS	Disease-free survival
EGF	Epidermal growth factor
EGFR	Epidermal growth factor receptor
EMT	Epithelial–mesenchymal transition
ERK	Extracellular regulated protein kinase
ESCC	Esophageal squamous cell carcinoma
ECM	Extracellular matrix
FAB	Focal adhesion binding
FAK	Focal adhesion kinase
FZD	Frizzled
GAP	GTPase-activating protein
GC	Gastric cancer
GEF	Guanine nucleotide exchange factor
GIST	Gastrointestinal stromal tumor
GSK3β	Glycogen synthase kinase 3 beta
HCC	Hepatocellular carcinoma
HEK	Human embryonic kidney
HFF	Human foreskin fibroblast
HNSCC	Head and neck squamous cell carcinoma
IHC	Immunohistochemistry
LEF	Lymphoid enhancer-binding factor
LNM	Lymph node metastasis
LUAD	Lung adenocarcinoma
MET	Hepatocyte growth factor receptor
MIBC	Muscle-invasive bladder cancer
mTOR	Mechanistic target of rapamycin
NSCLC	Non-small-cell lung cancer
OS	Overall survival
PDAC	Pancreatic ductal adenocarcinoma
PFS	Progression free survival
PM	Peritoneal metastasis
PTB	Phosphotyrosine-binding
PTC	Papillary thyroid carcinoma
PTP	Protein tyrosine phosphatase
RCC	Renal cell carcinoma
RFS	Relapse-free survival
RhoA	Ras homolog family member A
RTK	Receptor tyrosine kinase
SAM	Sterile alpha motif
SH2	Src homology 2
TCF	T cell factor
TCGA	The Cancer Genome Atlas
UALCAN	The University of ALabama at Birmingham CANcer data analysis Portal
YAP	Yes-associated protein
